# Durability of Concrete with Partial Replacement of Portland Cement by Incorporating Reactive Magnesium Oxide and Fly Ash

**DOI:** 10.3390/ma16072670

**Published:** 2023-03-27

**Authors:** Lucas Sequeira, Javier Forero, Miguel Bravo, Luís Evangelista, Jorge de Brito

**Affiliations:** 1 CERIS—Civil Engineering Research and Innovation for Sustainability, Department of Civil Engineering, Architecture and Georesources, Instituto Superior Técnico (IST), University of Lisbon, 1049-001 Lisbon, Portugal; 2 Postgraduate Program in Structural Engineering and Construction (PECC), Predio SG-12 Campus Darcy Ribeiro, University of Brasília, Brasilia 70910-900, Brazil; 3 CERIS—Civil Engineering Research and Innovation for Sustainability, Lisbon’s Polytechnic Engineering Institute (ISEL-IPL), 1959-007 Lisbon, Portugal

**Keywords:** concrete, MgO, fly ash, durability properties

## Abstract

In this research, the durability performance of sustainable concrete with the incorporation of reactive magnesium oxide (MgO) and fly ash (FA) was evaluated. The partial replacement of cement with these two materials is an appealing solution for the construction sector due to sustainability benefits and shrinkage reduction. The incorporation of FA by partial replacement of cement was carried out at 0%, 15% and 30%. The incorporation of MgO in concrete was carried out at 0%, 5%, 10% and 20%. Two types of MgO were used, one from Australia and another of Spanish origin. These two materials were evaluated in terms of their individual incorporation, and then an evaluation was carried out when the two were simultaneously used. In terms of durability, performance losses between 3% and 95% were obtained in all tests (water absorption by capillarity and immersion, carbonation depth and resistance to chloride penetration). However, over time, the difference in performance relative to the reference concrete tends to decrease due to the slow hydration that characterizes these two alternative materials. It was found that, in most of the tests, no overlapping of the negative effects occurred. In other words, the simultaneous incorporation of MgO and FA caused performance losses lower than the sum of the losses of their individual incorporation.

## 1. Introduction

The world population has been increasing exponentially in recent decades. One of the consequences of this population increase will be an increase in the number of dwellings and infrastructures, especially in large cities.

The construction industry will play a fundamental role in allowing the correct accommodation of this large number of human beings. The same industry is responsible for the emission of 10% of the CO_2_ produced worldwide. More specifically, 7.4% of CO_2_ comes from cement production. Based on these facts, it is clear that concrete production has a high ecological footprint [1]. The use of special mortars is a way of mitigating the impact of climate change by using them as a carbon dioxide capture, utilization and storage technology [2]. With the aim of reducing this footprint, several alternatives have been proposed, among which comes the one that will be presented in this paper [3]. The use of mineral admixtures in concrete production can help in the resolution of this problem. In this research, the use of MgO and fly ash was studied.

The cracking of concrete can lead to a reduction in its durability and can also compromise the safety of structures. This can be particularly problematic in regions with harsh weather conditions or high-traffic areas where constant exposure to environmental stressors can weaken concrete. Another problem with Portland cement concrete is the high alkalinity of the mix, which can cause the corrosion of reinforcing steel, leading to potential structural damage. This issue has been of concern in the construction industry for several decades, as the reinforcement is essential for the structural stability of concrete structures. To address these problems, researchers have been studying the use of active magnesium oxide and fly ash as partial replacements for Portland cement.

Regarding MgO, it is possible to produce concrete with its incorporation, reducing CO_2_ emissions [4]. The use of reactive MgO as a partial replacement for Portland cement in concrete can reduce the amount of CO_2_ emitted for two major reasons. The first one is the lower calcination temperature, since the production of Portland cement involves heating raw materials, such as limestone and clay, to temperatures of around 1450 °C. This process releases large amounts of CO_2_ into the atmosphere. In contrast, reactive MgO can be produced at much lower temperatures, typically around 750 °C, which results in much lower CO_2_ emissions [5,6,7]. That reduction may be explained by carbonation; i.e., when concrete is exposed to the atmosphere, it naturally absorbs CO_2_ over time. The use of reactive MgO can accelerate this process, as MgO reacts with CO_2_ to form magnesium carbonate. This reaction helps to offset the carbon emissions associated with the production of Portland cement. In this investigation, light-burned MgO was used, characterized by high reactivity, caused by its calcination temperature between 700 °C and 1000 °C [5]. These temperatures are relatively low compared to that needed for the production of Portland cement, which is 1450 °C [6,7].

Jin and AL-Tabbaa [5], citing Kramer and Shand [8,9], stated that much of the MgO used in industry is produced through the calcination of MgCO_3_, as seen in Equation (1).

MgCO_3_ + heat ⇒ MgO + CO_2_ (1)

Mo et al. [10] stated that in the hydration reaction of MgO brucite, Mg(OH)_2_, is obtained as the end product (Equation (2)).

MgO + H_2_O ⇒ Mg(OH)_2_ (2)

Du [11] presented more information on the hydration of magnesium oxide. The author states that the formation of brucite causes expansion in concrete because the hydration products of MgO have a larger volume than the reactants. One of the most important characteristics in the use of MgO is its reactivity. This property is evaluated through the reaction of MgO with water and dilute acids, and the reactivity depends mainly on the physical characteristics and purity of the material. The reactivity of MgO tends to increase with the reduction in its particle size and with the increase in its specific surface area. These two parameters are controlled by the production conditions, i.e., raw material purity and calcination temperature [12].

The lower the reactivity of a MgO sample, the slower its hydration. The smaller specific surface area causes more clumping of the particles in a sample, which leads to lower hydration. Jin and Al-Tabba and Mo et al. [5,10] reached the same conclusions.

One of the most important concrete characteristics is porosity. Given its relevance, several studies have already been conducted. Mo and Panesar [13] studied the porosity in cement pastes with MgO. The amount of MgO present was 0%, 10%, 20% and 40%. The researchers found that incorporating 20% MgO could lead to a 32% decrease in pore volume at 28 days when compared to that of the reference cement paste under accelerated carbonation. On the contrary, a 6–10% increase in pore volume was found in pastes with 10–40% MgO under non-carbonation conditions, indicating that the higher the amount of MgO used, the higher the porosity of concrete, impairing some of its properties in terms of durability [14].

Liu et al. [15] reached the same conclusions, where the use of 5% MgO as a substitute for OPC, in mortar production, led to a 19% increase in porosity, compared to the reference mortar, at 91 days.

Several studies have been conducted on cementitious materials with the incorporation of MgO. In terms of durability, the conclusions obtained by the different authors were clear.

Bravo et al. [16] analyzed the water absorption by capillarity in mortars with the presence of 5%, 10%, 15%, 20% and 25% MgO in their constitution. The authors stated that the greater the amount of MgO present, the greater the water absorption by capillarity relative to the reference mortar. In fact, when adding 25% MgO, the worst results were obtained, with an increase of about 44% in absorption.

On the other hand, Mavroulidou et al. [17] studied the water absorption capacity in concrete with the incorporation of 5% and 10% MgO, together with FA and metakaolin, as a substitute for OPC, at 28 days of age. It was found that the incorporation of 5% MgO caused a decrease in water absorption capacity, which was attributed to the better compaction of mixes with higher levels of MgO and metakaolin, due to the greater water requirements in its composition to maintain consistency. However, with the presence of 10% MgO, better results were obtained than those observed for the reference concrete, but worse results were obtained than those observed for concrete with 5% MgO.

Pu and Unluer [18] analyzed the carbonation capacity of concrete with the incorporation of 10% MgO, at 14 days. The authors observed that the carbonation depth in specimens with MgO was twice that obtained in the reference concrete. Gonçalves et al. [19] reached the same conclusions, observing an increase in the carbonation depth in mortars as the percentage of MgO increased. In fact, the presence of 20% MgO in the produced mortars increased the carbonation depth by between 139% and 483% at 91 days, depending on the reactivity of MgO used.

In summary, the use of MgO in cementitious materials leads to worse characteristics in terms of its durability. Analyzing the studies presented, it is clear that the presence of MgO causes an increase in water absorption by capillarity and immersion. The presence of this oxide also causes a greater depth of carbonation in specimens with MgO. Regarding the resistance of chloride penetration, taking into account the variation in the properties mentioned, it is expected that the presence of MgO causes a decrease in this capacity.

The use of fly ash is interesting because it allows for its recycling. This material is mainly composed of SiO_2_ and Al_2_O_3_. According to the American Society for Testing Materials [20], the fly ash used in this investigation is class F since the amount of SiO_2_ + Al_2_O_3_ + Fe_2_O_3_ (silicate + alumina + iron oxide) is higher than 70%. Physically, it is possible to observe a filler effect, which consists in filling the voids between particles of Portland cement with fly ash particles, because these have smaller dimensions [4]. Due to the presence of siliceous compounds, which in the presence of water can be related to calcium hydroxide, there is an increase in calcium silicate hydrate (C-S-H), leading to advantages in terms of durability [21,22].

During the first 24 h, fly ash has a retarding impact on concrete hydration. This phenomenon is because the ash needs the glassy fraction to solubilize in order to interact with the calcium hydroxide. After 28 days of age, hydrate growth occurs on the surface of the fly ash particles. The degree of hydration can increase up to 16%, in order to compensate for the lower degree of hydration at the beginning of curing [23].

Saha [24] demonstrated that the capillary water absorption in concrete with 10%, 20%, 30% and 40% FA was decreased by 4%, 13%, 29% and 32%, respectively, relative to the reference concrete at 28 days of age. The study noted that FA concrete lost capillary water absorption for two distinct reasons. The first is that FA has a greater specific surface area than cement, and the second is that FA reduces the thickness of the interfacial transition zone (ITZ) between aggregates and binders. Nayak et al. [25] concluded that water absorption by immersion and capillarity in concrete with 40% FA was 26% lower than that in the reference concrete, which is probably due to its improved reaction with the products released during the hydration process. The secondary gel or extra C-S-H produced in the presence of fine FA particles filled some pores inside the concrete, making it denser and more compact. Consequently, the permeability coefficient was reduced as the FA content increased to a given level [26].

Again, Saha [24] concluded that the chloride penetration at 28 days in concrete with 10%, 20%, 30% and 40% FA was reduced by 3%, 27%, 48% and 53%, respectively, relative to the reference concrete. The study suggested that this decrease is due to the fineness of fly ash. However, the lower concentration of alkali ions (Na^+^ and K^+^) and associated hydroxyl ions (OH^−^) in the pore solution is mentioned as another reason for this better behavior [27]. Sadrmomtazi et al. [28] reported an increase in chloride penetration at 28 days in concrete with FA compared to control concrete. On the other hand, at the age of 90 days, the chloride penetration decreased by up to 30% with the use of FA, due to the progress of pozzolanic reactions.

An experimental study by Turk et al. [29] showed that as the FA content in self-compacting concrete increases, the carbonation resistance decreases [30]. This is because the use of FA reduces the concentration of carbonation-prone products (calcium hydroxide and C-S-H). As a result, total CaO decreases due to higher carbonation rates [31].

With an increasing amount of FA in concrete, it is expected that water absorption by capillarity and immersion will show lower values than those of the reference concrete. The same happens for chloride penetration resistance and carbonation depth, as found in the studies presented above.

Analyzing the combination of fly ash with MgO, it was possible to conclude that MgO is insoluble with C-S-H from the hydration of Portland cement and fly ash. Thus, brucite will react with the silica from fly ash, forming a compound called hydrated magnesium silicate (M-S-H) [32]. Depending on the amount of silica present, the hydration of MgO may give rise to two products that may or may not coexist: brucite and M-S-H.

Choi et al. [33] focused their study on concrete with partial incorporation of FA and MgO. For the experimental campaign, they incorporated 20% FA in all mixes, while the amount of MgO varied between 0% and 5%. The specimens were water cured for 28 and 360 days. The authors used different water/binder ratios of 0.65 and 0.48. From the chloride ion migration test results, 65-M0-28 had a 1.1% lower coefficient than 65-M5-28, but 65-M0-360, with a longer curing period, had a 20% higher coefficient compared to 65-M5-360. In addition, MgO worked similarly in concrete with *w*/*b* = 0.48.

In the same study, the authors also analyzed the carbonation capacity of the different mixes produced at 28, 90 and 180 days of age. The carbonation depths of 65-M0-28 and 65-M5-28 were almost identical up to 180 days of exposure, with a difference of only 4%. On the other hand, 65-M5-360, having been cured for a longer duration, produced a shallower depth of carbonation (15%) than 65-M0-360 from the start. It is then possible to conclude that the carbonation depth at early ages is little affected by the presence of MgO and FA, showing better results at later ages. It is known that an increase in porosity promotes concrete carbonation since it facilitates the influx of CO_2_ [27,30,31].

In this experimental campaign, concrete with 0%, 5%, 10% and 20% MgO and 0%, 15% and 30% fly ash was produced. It should be noted that the percentages of incorporation are defined in terms of mass. Furthermore, the study focused mainly on the simultaneous use of these two materials, but this replacement will also be analyzed individually to better understand their combined action.

Two different types of MgO were studied, one of Australian origin (MgO-A) and the other from Spain (MgO-S), in order to obtain conclusions on how some specific characteristics of MgO influence the future performance of concrete. Durability-related characteristics were analyzed. An analysis of the concrete in its fresh state was also performed, by studying its workability. In terms of durability, the tests performed were water absorption by capillarity and immersion, carbonation depth and resistance to chloride penetration.

Nowadays there is not enough literature to allow the characterization of concrete with MgO and FA in terms of durability. It is important to fill this gap in order to be able to advance in the research of more sustainable concrete. Therefore, this is the main objective of this study. In fact, the scarce research conducted on the combined use of these two materials does not comprehensively assess the durability of these mixes. On the other hand, the evaluation of the use of MgO’s with different reactivity (a low-reactivity MgO and a high-reactivity MgO were used) in these concrete mixes with MgO and FA is also completely innovative.

## 2. Materials and Methods

### 2.1. Materials

For this investigation, the following materials were used: fine aggregates, coarse aggregates, cement CEM I 42.5R, tap water, MgO and fly ash. No additives were used in this experimental campaign.

Portland cement CEM I 42.5R was produced by Secil (Lisbon, Portugal). In terms of natural aggregates, two types were used: coarse aggregates (with commercial grading conforming to the designations 2/6, 6/12 and 12/20 of EN 12620 [34]) and siliceous sands (0/2 and 0/4). A class F fly ash was obtained from EDP-Gestão da Produção de Energia, S.A., at the Sines Power Plant, in Portugal (with 58% SiO_2_ and 24% Al_2_O_3_). Two different types of MgO were used, Spanish and Australian, with purities of 85.0% and 98.8%, respectively. In terms of specific surface area, MgO-S had 4.9 m^2^/g, while MgO-A had 51.2 m^2^/g. The reactivity was 3544 s and 14 s for the Spanish and Australian MgO’s, respectively. Liska et al. [12] also demonstrated that the reactivity of MgO tends to increase with the increase in the specific surface area. The fly ash used to produce concrete was also supplied by Secil. In Table 1, the particle size of the different binders is provided. Table 1 shows that particles of MgO-A clearly have a smaller size than particles of MgO-S.

### 2.2. Composition of the Mixes

In Table 2, the composition of the reference concrete is provided. Only the composition of the RM mix is provided because the others are the same.

Following a methodology presented by Nepomuceno et al. [35], the composition of each mix was determined. In order to characterize the reference concrete, a strength class C30/37 and a consistency class S2 were defined. Different water/binder ratios were used in order to maintain the S2 consistency class in all the mixes.

The only change in the composition of the other mixes is the amount of each binder, according to defined and explained percentages. In total, 21 concrete mixes were produced: reference concrete (RC); mixes with replacement ratios of 5%, 10% and 20% MgO-A and MgO-S, by mass; mixes with incorporations of 15% and 30% fly ash, by mass (with or without MgO). Each mix is identified as Cx/y:FA/MgO-A or Cx/y:FA/MgO-S, where x is the amount of FA and y is the amount of Australian or Spanish MgO, respectively.

### 2.3. Tests

It was decided to divide the experimental campaign into three phases. Firstly, the different materials that constituted concrete were evaluated. Then, the concrete mixes were analyzed in their fresh and hardened states.

In order to classify the different mixes in terms of durability, the tests previously mentioned were performed. The water absorption by capillarity was evaluated at 28 and 91 days, in 3 specimens, according to LNEC E-393 (1993) [36]. Water absorption by immersion was measured at 28 days, in 3 specimens, based on LNEC E-394 (1993) [37]. For chloride penetration resistance, the diffusion coefficient test was performed at 28 and 91 days, in 3 specimens for each age, according to NT BUILD 492 (1999-11) [38]. Finally, the carbonation depth was measured at 7, 28, 56 and 91 days of age, based on LNEC E391 (1993) [39]. All specimens were placed in a humid chamber for 28 or 91 days.

## 3. Results and Discussion

### 3.1. Consistency

**Figure 1:**
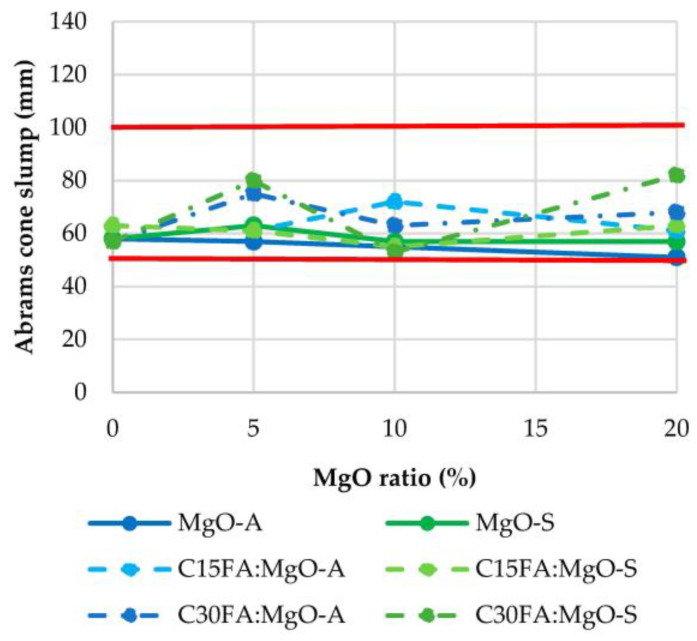
Abrams cone slump.

**Figure 2:**
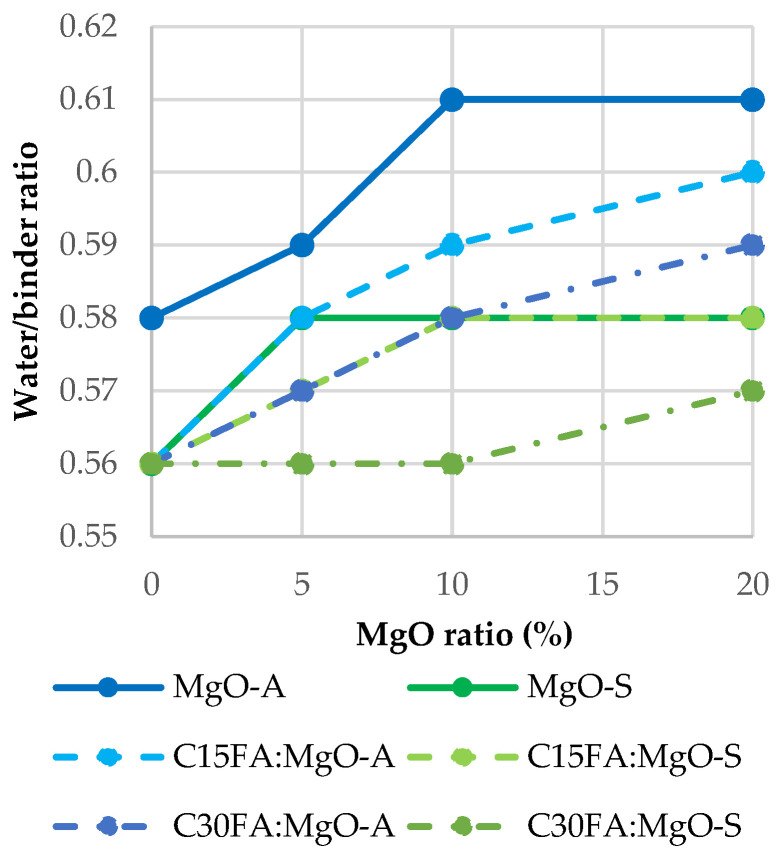
Water/binder ratio.

By increasing the incorporation ratio of reactive MgO, the consistency of the mixes tended to decrease. Therefore, it was necessary to increase the water/binder ratio in the mixes as reactive MgO increased to maintain the target consistency, as observed in Figure 1. Figure 2 shows that all mixes are within the S2 consistency (50–100 mm) class. Comparing the two different MgO’s (from Spain and Australia), a higher amount of water was required in the mix with MgO from Australia. This is due to the higher fineness and specific surface area of the MgO-A particles, since the greater their specific surface area, the greater the amount of water required absorbed by their surface. On the other hand, there was a slight decrease in the amount of water required in concrete with FA.

### 3.2. Water Absorption by Capillarity

**Figure 3:**
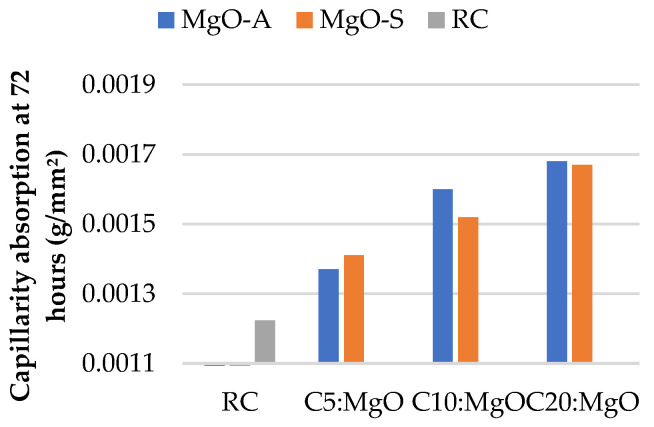
Water absorption by capillarity at 72 h versus MgO content, at 28 days.

**Figure 4:**
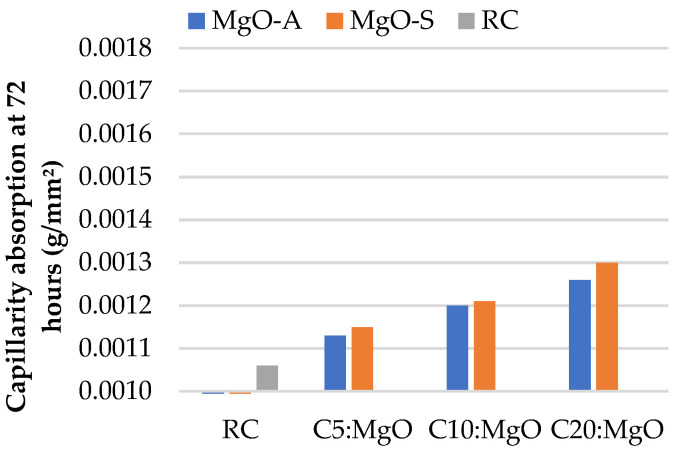
Water absorption by capillarity at 72 h versus MgO content, at 91 days.

Firstly, the results of water absorption by capillarity at 28 and 91 days for concrete with the incorporation of MgO are presented in Figure 3 and Figure 4, respectively. With the presence of MgO, the water absorption by capillarity tends to increase considerably. In fact, the greater the amount of MgO incorporated, the greater the water absorption. It was possible to obtain, especially at 91 days, better results when MgO-A was used. This phenomenon can be due to the smaller specific surface area of the particles of MgO-S leading to greater porosity.

As expected, the capillarity values at 28 days are higher than those at 91 days. An increase of 37% is observed in C20:MgO-A, relative to RC, at 28 days. However, this increase is only 23% at 91 days. Choi et al. [33] reached the same conclusion and explained that this phenomenon occurs due to the delayed hydration of MgO relative to Portland cement type I. The same authors found that the porosity of concrete with MgO tends to decrease over time. This conclusion is a very important fact in the study of the durability characteristics of concrete produced with MgO.

**Figure 5:**
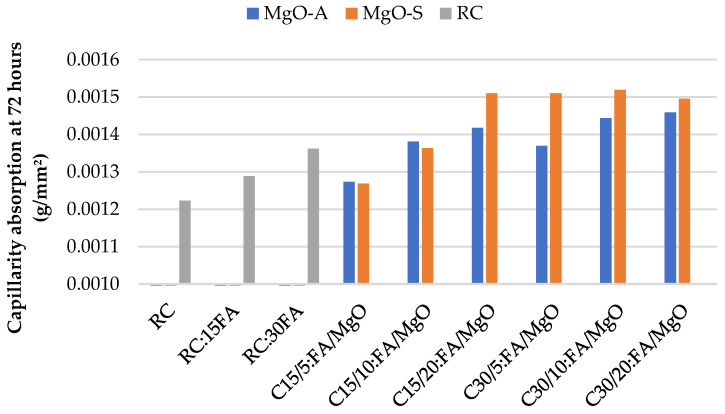
Capillarity absorption at 72 h in mixes with MgO and FA at 28 days.

**Figure 6:**
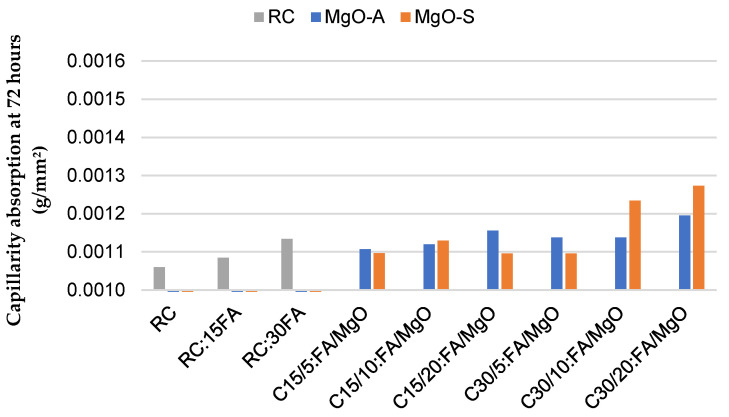
Capillarity absorption at 72 h in mixes with MgO and FA at 91 days.

Analyzing Figure 5 and Figure 6, it is possible to observe the water absorption by capillarity in concrete with MgO and FA at 28 and 91 days. By incorporating MgO and FA in the constitution of concrete, it is concluded that, as the amount of these two materials increases, the capillary absorption capacity also tends to increase. Again, at 28 days, this same capacity shows higher values than the tests performed at 91 days. The best results were obtained in the mix C15/5:FA/MgO, with an increase of about 4%, relative to RC, both at 28 and 91 days. Again, MgO-A seems to show better results than the Spanish counterpart. Observing Table 3, it is noticeable that, when these two materials are used simultaneously, the results obtained were always better than the expected theoretical values (sum of the effects of the individual placement of these elements). This is quite easy to observe when, at 91 days, the RC:15FA mix presents similar values when MgO is added. Thus, in terms of this property, the simultaneous use of MgO and FA in the production of concrete seems to be interesting.

### 3.3. Water Absorption by Immersion

**Figure 7:**
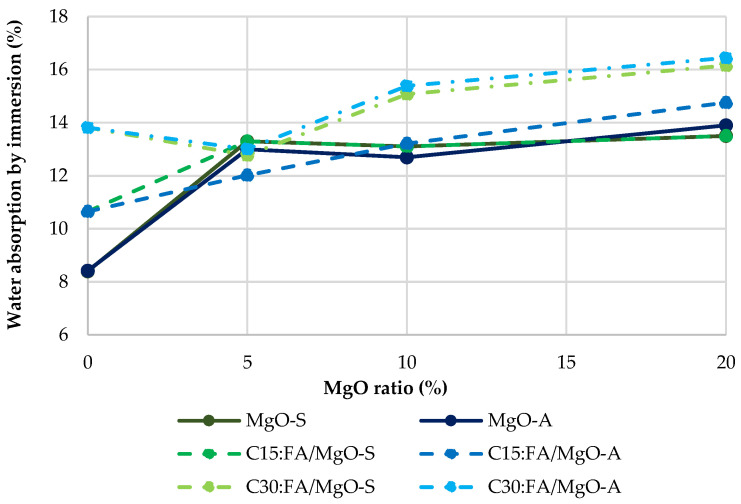
Water absorption by immersion at 28 days.

Figure 7 shows that the water absorption by immersion at 28 days tends to increase as the incorporation ratio of MgO and FA increases. Using only MgO-A, the variation varies between increases of 54% and 65% with respect to RC for the use of 5% and 20% MgO-A, respectively. Similar values were obtained for the Spanish counterpart. The worst results were seen in the C30:FA/MgO mixes, as expected, with an absorption of about 16% for both MgO’s, almost doubling the RC value. However, it should be noted that the combined use of these materials (MgO and FA) shows an improvement relative to the expected theoretical value. This can be proved by analyzing, for example, the C15/10:FA/MgO-A mix. The RC15:FA mix shows an increase in the water absorption of 26.5%, in relation to RC. The C10:MgO-A mix presents an increase of 51%. It would then be expected that the joint use would cause a 77.5% increase in the absorption capacity compared to RC. However, this increase was only 57%. When MgO is mixed with water, it undergoes a chemical reaction to form magnesium hydroxide, which can fill the pore spaces in the concrete and reduce water absorption. Physically, the use of FA can originate a filler effect, which consists in filling the voids between particles of Portland cement with fly ash particles, because these have smaller dimensions [4]. When MgO and fly ash are used simultaneously in concrete, they complement each other’s properties. MgO fills the pore spaces, while fly ash produces additional C-S-H gel and improves durability. As a result, the combination of these two materials provides better results in terms of water absorption by immersion than the sum of the individual effects [32].

### 3.4. Carbonation

**Figure 8:**
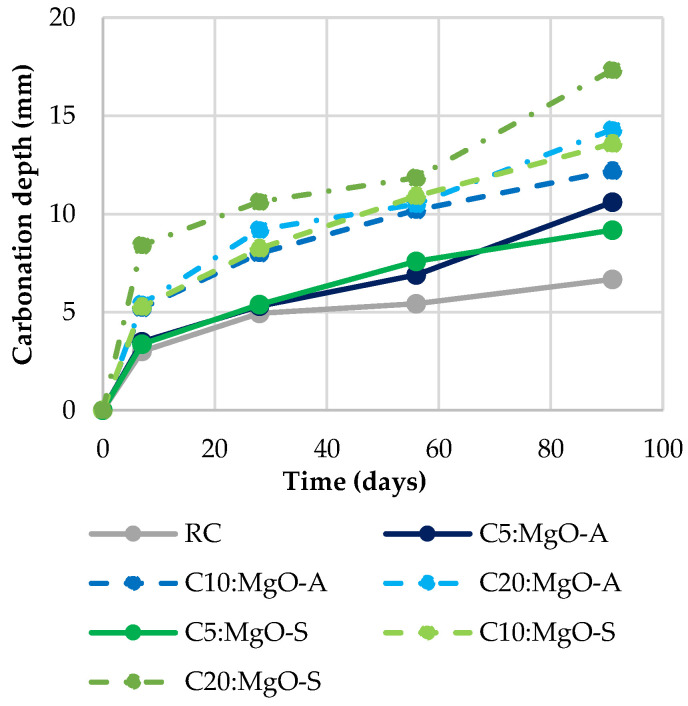
Carbonation depth in mixes with MgO.

**Figure 9:**
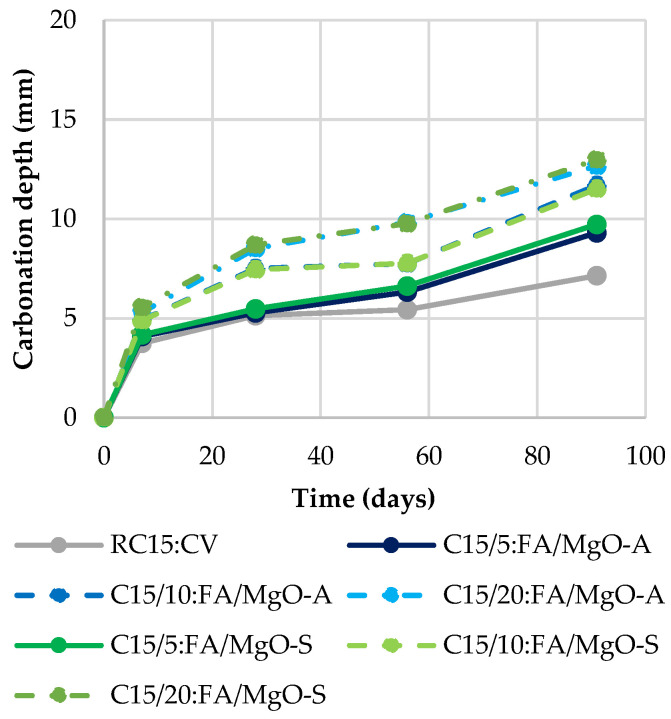
Carbonation depth in mixes with MgO and 15% FA.

**Figure 10:**
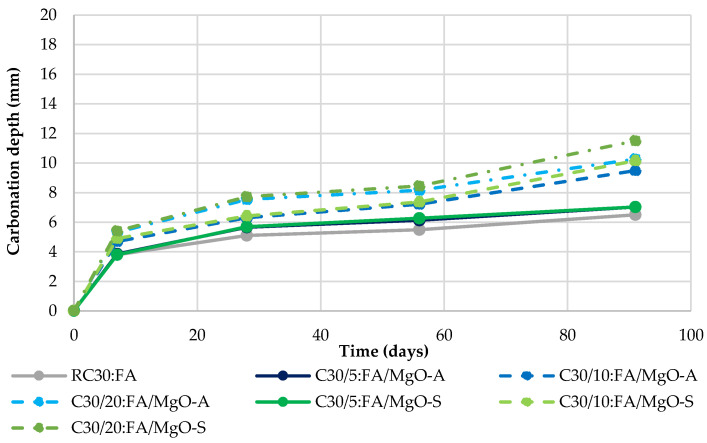
Carbonation depth in mixes with MgO and 30% FA.

In Figure 8, Figure 9 and Figure 10, the carbonation depth at 7, 28, 56 and 91 days of concrete mixes that were cured in a chamber with 5% CO_2_, 60% RH and 23 °C temperature is provided. It is immediately clear that with the incorporation of MgO and FA, the carbonation depth increases. In fact, when the ratio of these materials used in the mixes increases, the carbonation tends to present higher values. This phenomenon was expected due to several reasons. First, the increase in the water/binder ratio in the mixes with MgO and/or FA causes a more porous matrix.

On the other hand, the partial replacement of Portland cement causes lower production of C-S-H and Ca(OH)_2_, in which interaction between them would lead to a decrease in the progress of carbonation. In addition, according to Gonçalves et al. [19], the decreased quantity of Ca(OH)_2_, which exhibits a pH level of ~12.5, and a higher amount of Mg(OH)_2_, with a pH of ~10.5, means that the overall pH level lowers, thereby showing an increased rate of carbonation according to the phenolphthalein test. This, together with the existence of a more porous microstructure, relative to RC, will be quite coherent justifications.

The MgO from Spain shows worse results than the one from Australia. As previously mentioned, this is because the lower specific surface area of the MgO-S particles causes greater porosity, facilitating the progression of carbonation to greater depths. This can be seen in Figure 8, as the use of 20% MgO-A increases the carbonation depth at 91 days by 114% relative to RC, while in MgO-S, this increase exceeds 160%.

Table 4 shows the results for the mixes with MgO and FA. Once again, it is important to note that the simultaneous use of these two materials presents very good results, in comparison with the summed effects of their individual incorporation. It was possible to observe that in 11 of the 12 concrete mixes produced with MgO and FA, better values of this property than the expected theoretical values were obtained.

Knowing that the presence of FA leads to an improvement in carbonation capacity [24,30], it should be noted that the best results were obtained when a mixture of 30% FA with MgO was used (a decrease in carbonation depth of 2.5% appears in RC30:FA, compared to RC). This is because the fly ash particles seem to be able to fill the interstitial pores (filler effect), halting the advance of carbonation depth. In addition, the use of FA reduces the concentration of carbonation-prone products (CH and CSH). As a result, total CaO decreases due to higher carbonation rates [31].

### 3.5. Resistance to Chloride Penetration

**Figure 11:**
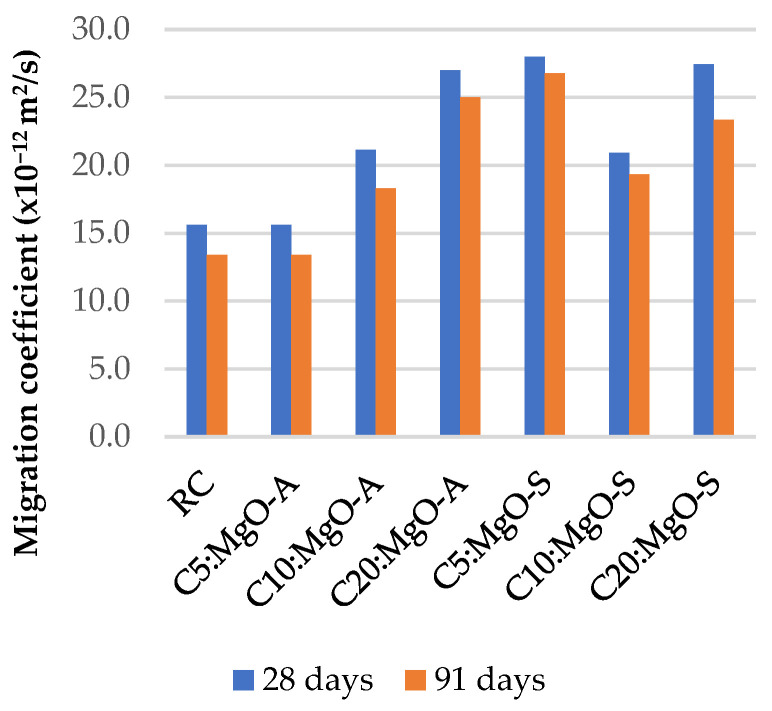
Chloride ion migration coefficient in mixes with MgO.

**Figure 12:**
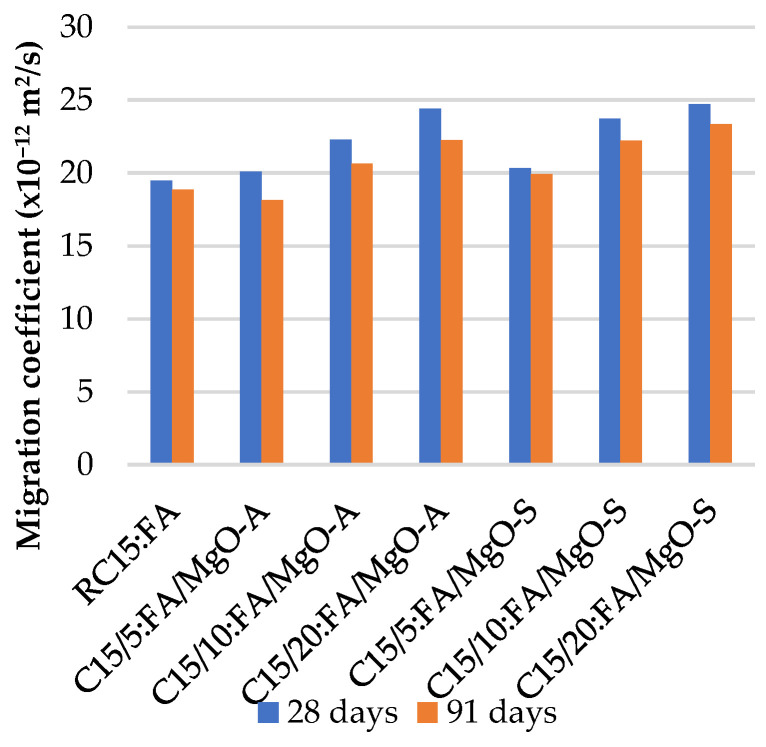
Chloride ion migration coefficient in mixes with MgO and 15% FA.

**Figure 13:**
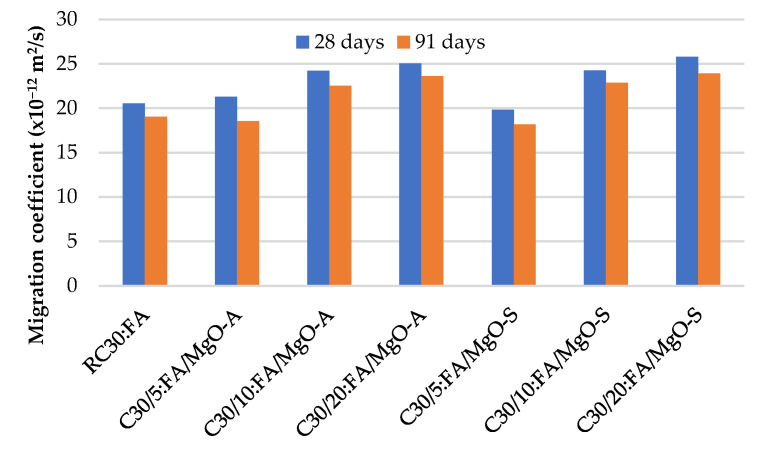
Chloride ion migration coefficient in mixes with MgO and 30% FA.

Figure 11, Figure 12 and Figure 13 summarize the chloride penetrability test results at 28 and 91 days. As expected, it is evident that with the incorporation of MgO and FA, the chloride ion migration coefficient increases.

The worst results were obtained when 20% MgO was used, with an increase of 100% in the chloride ion penetration coefficient relative to RC. As with the previous properties, the theoretical value expected for the combination of MgO and FA is substantially higher than the value obtained in the laboratory, as shown in Table 5. This means that the simultaneous use of MgO and FA in concrete also has positive consequences on these concrete properties. As expected, the coefficients at 91 days present lower values than those at 28 days. This means that there is a small improvement in the resistance to chloride penetration over time.

**Figure 14:**
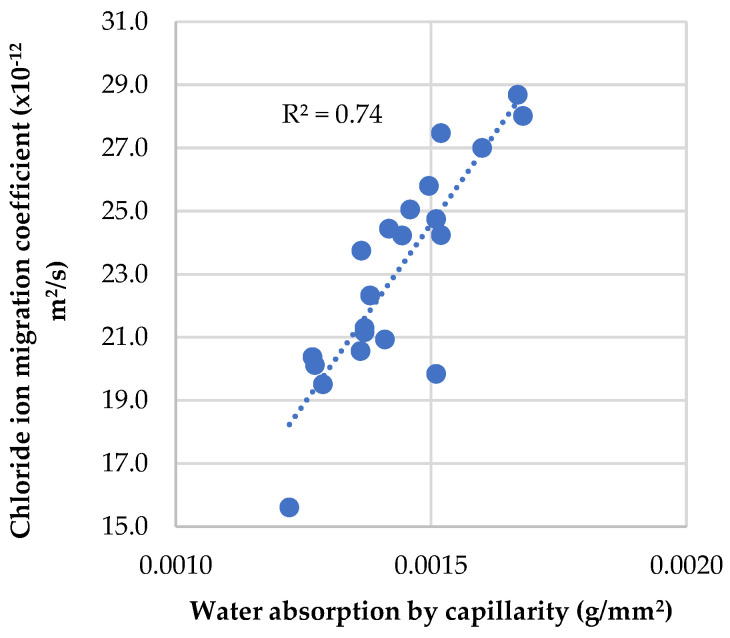
Relationship between chloride ion migration coefficient and water absorption by capillarity.

Several studies have been carried out comparing chloride ion penetration with water absorption by capillarity. Ferreira [40] concluded that the chloride ion diffusion coefficient and the water absorption by capillarity have a linear relationship. As seen in Figure 14, the coefficient of determination at 28 days was 0.74, which shows a reasonable linear relationship between both properties in the concrete mixes evaluated in this investigation.

**Figure 15:**
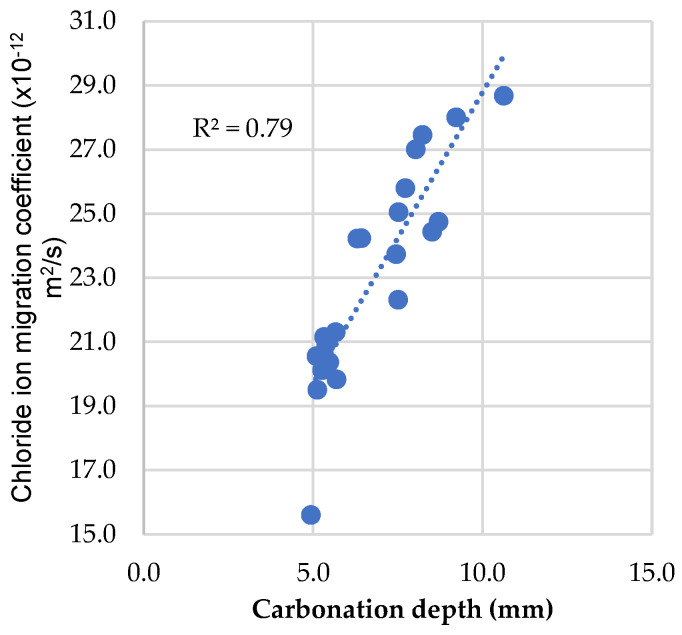
Relationship between chloride ion migration coefficient and carbonation depth.

The linear relationship between the carbonation depth and the chloride diffusion coefficient was also analyzed, at 28 days, and it can be observed in Figure 15. In this case, the linear regression coefficient was 0.79, also indicating a reasonable linear relationship between the mentioned properties in mixes with MgO and FA. The linear relationships observed allow demonstrating that the mentioned properties are influenced by identical factors, both in conventional concrete and in concrete with partial incorporation of MgO and/or FA.

### 3.6. Comparative Analysis between Concrete Durability and Compressive Strength

It is important to compare the compressive strength with the different durability properties analyzed in order to understand whether there is a relationship between the different properties. For the reference concrete, a compressive strength of 44.2 MPa was obtained at 28 days, and a compressive strength of 48.3 MPa was obtained at 91 days. In Table 6, the variation in the properties mentioned in the ternary mixes (OPC, MgO, FA), relative to the reference concrete, is provided.

Regarding compressive strength, it is clear that there is a decrease in strength capacity in concrete with partial incorporation of MgO and FA. In fact, the greater the amount of MgO and FA incorporated, the greater the observed reduction in this capacity compared to the reference concrete. At 28 days, there were decreases of between 23% and 60% in the compressive strength of ternary mixes compared to RC. However, at 91 days of age, this decrease ranged only between 13% and 40%, proving the better behavior of these concrete mixes with the progress of curing time.

It is clear that the decay in the mechanical capacity of concrete is accompanied by worse results in terms of durability. The simultaneous presence of MgO and FA in concrete composition leads to worse results in all durability properties studied. As the amount of these materials increases, a worse behavior of ternary mixes is also observed.

It is concluded that the mechanical and durability properties present similar behavior in concrete with MgO and FA. This phenomenon is proved by verifying that with increasing amounts of MgO and FA in the cement matrix of concrete, worse compressive strength results are obtained. The same was found for durability.

## 4. Conclusions

This research served to study the influence, in terms of durability, of concrete with partial incorporation of MgO and fly ash. It was possible to analyze the corresponding variations when Portland cement was replaced with 5%, 10% and 20% MgO and/or 15% and 30% fly ash. In almost all the properties, worse results were obtained with the presence of one of these materials, or both, relative to the reference concrete.

It was also found that Spanish MgO generally presents worse results when compared to the Australian counterpart. This was due to its lower specific surface area and, consequently, to its lower reactivity. These characteristics increase the porosity of the cement paste. It is well known that the porosity of the cement paste is of vital importance in determining the durability behavior of concrete.

By incorporating MgO and FA in the constitution of concrete, it is concluded that as the amount of these two materials increases, the capillary absorption capacity also tends to increase. For mixes with simultaneous incorporation of MgO and FA, there was an increase in all mixes produced, ranging from 3.7% (C15/5:FA/MgO-S) to 24.3% (C30/10:FA/MgO-S), at 28 days of curing age.

A similar behavior was detected in the water absorption by immersion capacity at 28 days. The combined incorporation of the two materials caused worse results. In individual terms, the presence of only 15% FA causes a 26.5% increase in water absorption capacity. Concrete with 30% FA and 20% MgO-A shows very similar results, with an increase of 64% and 65% in this property, respectively, compared to conventional concrete. In the combined use of MgO and FA, the best result was obtained for the mix C15/5:FA/MgO-S, with an increase of 39.5% relative to RC.

Observing the results obtained for carbonation depth, similar results were obtained. The worst result was observed for C20:MgO-S, with an increase of 160% in carbonation depth at 91 days. However, the use of FA originated an improvement in comparison to RC. The lowest carbonation depth value was obtained for the mix RC30:FA.

As expected, with the incorporation of MgO and FA, the chloride ion migration coefficient increases. The worst results were obtained when 20% MgO was used, with an increase of 100% in the chloride ion penetration coefficient relative to RC. As previously mentioned, with the increase in the amount of MgO and FA in the cement matrix of concrete, there is an increase in the penetration of chloride ions, for all mixes, with the best value being reached when using 30% FA and 5% MgO, causing an increase of 35.4% in this property relative to RC.

By studying the compressive strength at 28 and 91 days, trends similar to those observed in the durability properties were found. The partial replacement of OPC causes a decrease in the mechanical capacity. Accompanying this decrease is a worse durability behavior of the non-conventional concrete. It is thus concluded that the presence of MgO and FA in concrete negatively affects the mechanical and durability characteristics of concrete.

Finally, one of the most important conclusions was that in none of these properties was there total overlapping of the negative effects. In the results obtained, an improvement is obtained when FA and MgO are combined, relative to the expected theoretical result. The theoretical value corresponds to the sum of the individual effects of each material. It was observed that in all the analyzed properties, the results obtained in ternary mixes were better than the expected theoretical results. This improvement was especially observed in the carbonation depth and resistance to chloride ion penetration.

Comparing the ratio of OPC replaced with the increase in the studied properties, it is possible to draw some conclusions. Only in the water absorption by capillarity, decreases with the use of MgO and FA, relative to RC, lower than the ratio of incorporation of these materials in the different mixes were observed. For example, in the mix C30/20:FA/MgO-S, the simultaneous incorporation of MgO and FA (in a total of 50%), originated an increase of only 20% in the water absorption by capillarity relative to RC. In the remaining properties, the opposite phenomenon was found. The exception is the mix C30/5:FA/MgO, where low values of carbonation depth were obtained.

In conclusion, it is clear that the combined use of MgO and FA shows better results than would be theoretically expected. However, except for water absorption by capillarity, the OPC replacement ratio is higher than the observed variation. The simultaneous use of these materials seems to work better, but more studies are needed to find the ideal incorporation ratio that could lead to better results. In this research, the incorporation of 15% FA and 5% MgO in concrete seems to be interesting. On the other hand, the replacement of 50% OPC seems excessive.

The practical benefits of using reactive magnesium oxide and fly ash as partial replacements for Portland cement in concrete production are significant. First and foremost, these materials are cost-effective and readily available, making them an attractive option for builders and engineers seeking to improve the sustainability and durability of their structures. Additionally, the use of active magnesium oxide and fly ash can improve the workability of concrete, making it easier to pour, mold and shape into the desired form. This can be particularly beneficial in construction projects where intricate or complex designs are required, such as in the construction of high-rise buildings, bridges and other infrastructure. From a maintenance perspective, the use of reactive magnesium oxide and fly ash can also improve the longevity and safety of concrete structures. By improving the resistance of concrete to cracking, structures can be expected to last longer and require less frequent repairs and maintenance.

The use of reactive magnesium oxide and fly ash as partial replacements for Portland cement in concrete production shows promising results. However, further research is needed to fully explore and optimize the potential of these alternative materials.

One area of further research is to investigate the performance of concrete mixes containing different ratios of reactive magnesium oxide and fly ash. This would help to identify the optimal mixture for achieving maximum performance and sustainability while maintaining cost-effectiveness. In addition, the long-term durability of concrete structures containing reactive magnesium oxide and fly ash should be studied. This would involve testing the performance of these structures under different environmental conditions over an extended period of time. This information would be valuable in determining the maintenance requirements of concrete structures containing these alternative materials. Another area of further research is to study the effects of using reactive magnesium oxide and fly ash in combination with other sustainable construction materials, such as recycled aggregates, in order to further reduce the environmental impact of concrete production. These research topics will be crucial to optimize the use of these materials in concrete structures with maximum performance, sustainability and cost-effectiveness for growing cities and communities.

## Figures and Tables

**Table 1:** Particle size of the different binders.

Material	<3 μm (%)	Between 3 and 32 μm (%)	>32 μm (%)
OPC	25.9	56.2	17.9
FA	21.3	55.9	22.8
MgO-S	12.1	15.3	72.6
MgO-A	40.1	56.0	3.9

**Table 2:** Composition of the reference concrete (RC).

Binder		0.098
Fine aggregates	0–0.063	0.001
	0.063–0.125	0.004
	0.125–0.25	0.030
	0.25–0.5	0.079
	0.5–1	0.097
	1–2	0.085
Coarse aggregates	2–4	0.036
	4–5.6	0.012
	5.6–8	0.050
	8–11.2	0.072
	11.2–16	0.153
	16–22.4	0.092
Water		0.174
Voids		0.017
Total		1.000

**Table 3:** Real values obtained in the laboratory and theoretical expected values at 28 and 91 days.

Mix	28 Days		91 Days	
	Δ Real (%)	Δ Expected Theoretical (%)	Δ Real (%)	Δ Expected Theoretical (%)
C15/5:FA/MgO-A	+4.1	+17.4	+4.4	+8.9
C15/5:FA/MgO-S	+3.7	+20.7	+3.4	+10.8
C15/10:FA/MgO-A	+13.0	+36.2	+5.7	+15.5
C15/10:FA/MgO-S	+11.5	+29.7	+6.5	+17.4
C15/20:FA/MgO-A	+16.0	+42.8	+9.0	+21.2
C15/20:FA/MgO-S	+23.5	+41.9	+3.4	+24.9
C30/5:FA/MgO-A	+12.0	+23.4	+7.3	+13.6
C30/5:FA/MgO-S	+23.5	+26.7	+3.3	+15.5
C30/10:FA/MgO-A	+18.0	+42.2	+12.8	+20.2
C30/10:FA/MgO-S	+24.3	+35.7	+16.4	+22.1
C30/20:FA/MgO-A	+19.3	+48.8	+17.1	+25.8
C30/20:FA/MgO-S	+22.3	+47.9	+20.1	+29.6

Note: Δ expected theoretical of mix CX/Y:FA/MgO corresponds to the sum of Δ real of mix RCX:FA and Δ real of mix CY:MgO.

**Table 4:** Real values obtained in the laboratory and theoretical expected values, at 91 days.

Mix	91 Days	
	Δ Real (%)	Δ Expected Theoretical (%)
C15/5:FA/MgO-A	39.6	65.5
C15/5:FA/MgO-S	46.0	44.9
C15/10:FA/MgO-A	75.5	90.4
C15/10:FA/MgO-S	72.7	110.9
C15/20:FA/MgO-A	90.1	121.6
C15/20:FA/MgO-S	94.9	167.2
C30/5:FA/MgO-A	5.4	37.1
C30/5:FA/MgO-S	5.4	35.2
C30/10:FA/MgO-A	42.4	80.8
C30/10:FA/MgO-S	52.4	101.3
C30/20:FA/MgO-A	54.0	111.9
C30/20:FA/MgO-S	72.5	157.6

Note: Δ expected theoretical of mix CX/Y:FA/MgO corresponds to the sum of Δ real of mix RCX:FA and Δ real of mix CY:MgO.

**Table 5:** Real values obtained in the laboratory and theoretical expected values, at 91 days.

Mix	91 Days	
	Δ Real (%)	Δ Expected Theoretical (%)
C15/5:FA/MgO-A	35.4	77.2
C15/5:FA/MgO-S	48.6	84.8
C15/10:FA/MgO-A	53.9	127.2
C15/10:FA/MgO-S	65.7	114.6
C15/20:FA/MgO-A	66.0	140.4
C15/20:FA/MgO-S	74.1	139.8
C30/5:FA/MgO-A	38.1	78.3
C30/5:FA/MgO-S	35.4	85.9
C30/10:FA/MgO-A	67.7	128.3
C30/10:FA/MgO-S	70.5	115.8
C30/20:FA/MgO-A	76.0	141.5
C30/20:FA/MgO-S	78.2	140.9

Note: Δ expected theoretical of mix CX/Y:FA/MgO corresponds to the sum of Δ real of mix RCX:FA and Δ real of mix CY:MgO.

**Table 6:** Mechanical and durability behavior of the different mixes.

Variation Relative to RC (%)						
Mix	Compressive Strength		Water Absorption by Capillarity	Water Absorption by Immersion	Carbonation Depth	Chloride Penetration
	28 Days	91 Days	91 Days	28 Days	91 Days	91 Days
C15/5:FA/MgO-A	−26.9	−15.5	+4.40	+42.7	+39.6	+35.4
C15/5:FA/MgO-S	−23.1	−13.7	+3.43	+39.5	+46.0	+48.6
C15/10:FA/MgO-A	−29.3	−15.5	+5.65	+57.0	+75.5	+53.9
C15/10:FA/MgO-S	−30.5	−17.3	+6.54	+65.4	+72.7	+65.7
C15/20:FA/MgO-A	−41.1	−34.5	+9.03	+75.3	+90.1	+66.0
C15/20:FA/MgO-S	−44.8	−36.8	+3.38	+82.1	+94.9	+74.1
C30/5:FA/MgO-A	−34.6	−15.0	+7.30	+54.4	+5.4	+38.1
C30/5:FA/MgO-S	−34.1	−18.9	+3.34	+51.7	+5.4	+35.4
C30/10:FA/MgO-A	−46.9	−29.1	+12.77	+82.8	+42.4	+67.7
C30/10:FA/MgO-S	−42.6	−26.2	+16.41	+79.9	+52.4	+70.5
C30/20:FA/MgO-A	−58.2	−39.5	+17.13	+95.3	+54.0	+76.0
C30/20:FA/MgO-S	−59.1	−38.5	+20.11	+91.9	+72.5	+78.2

## Data Availability

Not applicable.
